# Next‐Generation Therapies for Gastrointestinal Repair: Converging Traditional Medicine and Tissue Engineering

**DOI:** 10.1002/jgh3.70312

**Published:** 2025-12-17

**Authors:** Archna Dhasmana, Indra Rautela, Atul Singh, Ayushi Santhanam, Sumira Malik, R. K. Govindarajan, Subham Preetam

**Affiliations:** ^1^ Himalayan School of Biosciences, Swami Rama Himalayan University Dehradun Uttarakhand India; ^2^ Department of Biotechnology School of Applied and Life‐Sciences Dehradun Uttarakhand India; ^3^ G. B. Pant University of Agriculture and Technology Pant Nagar Uttarakhand India; ^4^ Amity Institute of Biotechnology Amity University Jharkhand Ranchi India; ^5^ University Center for Research & Development (UCRD) Chandigarh University Mohali Punjab India; ^6^ Department of Biotechnology Karpagam Academy of Higher Education Coimbatore Tamil Nadu India; ^7^ Centre for Natural Products and Functional Foods Karpagam Academy of Higher Education Coimbatore Tamil Nadu India; ^8^ Department of Chemical Engineering School of Engineering, Monash University Malaysia Bandar Sunway Selangor Malaysia

**Keywords:** bio‐engineered, cell, gastrointestinal tract, graft, regeneration, remodeling, ulcers

## Abstract

The gastrointestinal (GI) tract plays a vital role in regulating metabolic pathways, nutrient absorption, and cellular homeostasis. Abnormalities in the GI tract often arise from unhealthy lifestyles, genetic mutations, and prolonged medication use, leading to disorders such as ulcers, cirrhosis, and malignancies. Conventional treatments including chemotherapy and surgery remain limited by high costs, invasiveness, and incomplete tissue recovery. Recent advances in bioengineering have enabled the development of grafts and drug‐delivery systems that repair and regenerate functional GI tissue. This review presents a comprehensive overview of GI disorders, their current therapeutic options, and emerging tissue‐engineering strategies that integrate regenerative medicine and traditional therapies to address metabolic and genetic dysfunctions.

## 1 Introduction

The gastrointestinal (GI) tract regulates all metabolic pathways, including absorption, excretion, enzymatic regulation, and secretion. Partly or completely, dysfunction of the GI tract results in severe health issues and disorders [1]. To address these challenges, preventive strategies and sustainable therapeutic systems have been developed. Several studies have shown that distortion of the epithelial layer in the GI tract triggers regeneration through homeostasis, inflammation, and healing responses [2]. However, persistent inflammation can lead to functional loss and chronic conditions such as peptic ulcers and irritable bowel syndrome (IBS).

In developed countries like the US, Canada, and Australia, gastrointestinal disorders affect approximately 10% of the population, while the prevalence of irritable bowel syndrome (IBS) can reach 20% [3, 4]. The key factors lacking in these problems are non‐systematic pre‐diagnosis, limited donor tissue/organ, and improper clinical management. Severe GI problems require operative treatments or surgical resection of the damaged tissue and consequent transplantation in short bowel syndrome or organ damage [4].

**FIGURE 1:**
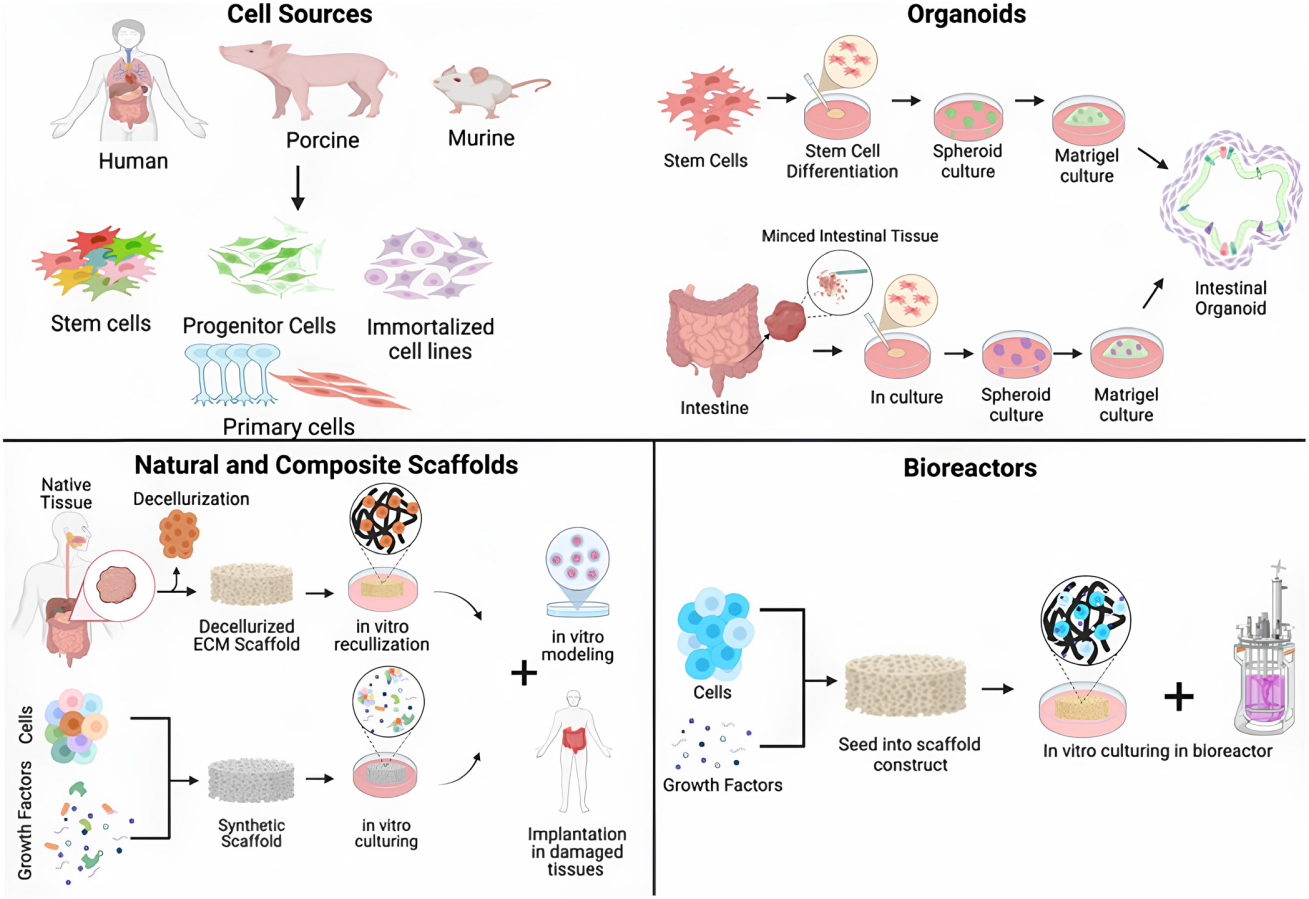
Present approaches to creating tissue‐engineered intestine focus on the strategic choice of GI cell sources, developing organoids with either natural or composite scaffold support, and utilizing bioreactor systems [5].

Prolonged medication and repeated surgeries often reduce treatment efficacy, increase economic burden, and lower patient survival rates. Consequently, researchers have turned to tissue engineering and regenerative medicine approaches to restore, repair, and remodel the defective GI tract. As summarized in Figure 1, recent advances in tissue‐engineered intestine models aim to create viable preclinical systems for studying drug response and developing regenerative therapies [5]. The bioengineered polymeric grafts, either acellular or cellular, are used as in vitro models to study the therapeutic effect of drugs or are implanted as tissue‐engineered grafts for regenerating damaged tissue. In vitro, bio‐engineered GI grafts are used as cost‐effective models designed explicitly for the toxicological, absorption, and cellular interaction studies concerning the animal models [6]. Based on these pre‐models, the applicability of the bioengineered GI grafts is evaluated at the preclinical and clinical levels as an economical treatment for the remodeling of the damaged tissue or organs, thereby improving the patients' lifestyle. Several attempts have been made at the preclinical and clinical levels to regenerate the GI defects by bioengineered grafts synthesized from natural, synthetic or bio‐synthetic polymers, composite, etc. There are specific regulating elements for tissue regeneration and repair through bio‐engineered grafts for implantation: the ultra‐structure of tissue matrix, surface functional groups for cellular signaling and multifaceted cellular alignment, and tissue flexibility as the native one [7].

At the cellular level, a well‐organized multicellular network on a biocompatible matrix is essential for engineering functional GI tracts. The matrix must closely mimic the native extracellular matrix (ECM) in composition, mechanical strength, flexibility, and porosity to support cell migration and vascularization [8]. Recent advances in GI graft fabrication highlight combinatorial strategies that integrate scaffold materials with tissue‐specific cells cultured in vitro [9]. Biocompatible and antimicrobial polymers have also been used to develop tubular grafts resistant to pathogens and capable of regenerating epithelial layers.

Natural biopolymers such as collagen, gelatin, chitosan, and silk are widely used for scaffold fabrication, while synthetic composites like PLA, PCL, and PLGA enhance mechanical stability [10, 11]. ECM‐derived proteins are particularly valuable due to their biocompatibility, biodegradability, and bioactivity, though acellular tissue grafts may carry risks of disease transmission. Emerging technologies such as bioprinting and nano‐matrix fabrication now enable large‐scale production of biomimetic grafts. Nanocomposites of collagen, chitosan, silk, PGA, and PLA have shown excellent potential for supporting cell growth and forming mechanically stable tubular organ models [12, 13]. Thus, this review discusses the GI tract tissue composition, functioning, deformities, treatments and innovative approaches to designing bio‐engineered functional grafts.

## 2 Anatomy and Abnormalities

### 2.1 Anatomy of the Gastrointestinal Tract

The GI tract is a structurally complex tubular system with diverse functions ranging from a transit tube (esophagus) to digestion (stomach), nutrient and water absorption (intestine), and excretion of waste (rectum) [14]. The tissue cellular layer of the GI tract comprises four types: *mucosa*, *submucosa*, *muscularis propria* and the *serosa*, connected with a neuro‐vascularized network [15]. The two layers *mucosa* and *muscularis propria* have a compact intercellular matrix, that is, epithelium, lamina propria, and muscular mucosae consist of mucosa and the internal circular muscle layer, intermuscular space, and superficial longitudinal muscle layer form *muscularis propria*. The core neural system in GI has a compact network of neurons called the enteric nervous system (ENS), which varies in different sections of GI and helps in muscular contraction and epithelial cell functioning. The neural network integrated with the muscular cellular layer helps to regulate food movement, digestion and the flexibility of tissue [16].

### 2.2 Disorder Associated With Physical Deformities of the Digestive Tract

The GI tract is sensitive to many diseases, which can be caused by parasites, fungi and other microbes and lead to bloating, ulcers, constipation, and other diseases [17]. Clinical screening of cardiovascular and abdominal regions detects systemic disorders and inflammation. Distension, auscultation, and palpation of the abdominal cavity directly affect the motility of the small and large intestines.

Propellant contraction in the cecum, ascending and ventral colon is responsible for the intestinal borborygmi and prolonged rushing sounds [18]. Similarly, postprandial distress syndrome is also a food‐induced distress. In epigastric pain syndrome and overlapping postprandial distress syndrome, epigastric pain and a high burning sensation are shown to become prominent during fasting or heavy meal ingestion [19]. FGID (functional gastrointestinal disorder), also known as a gut–brain interaction, is a much more common disorder (40%) than other GI tract disorders [20]. It includes many groups of disorders like fibromyalgia (chronic stage of pain), chronic fatigue syndrome, etc. [21]. In the chronic stage, severe pain and dyspepsia have been seen in patients.

IBS causes distress in the abdomen, alteration and bloating. On the other hand, functional dyspepsia causes epigastric aches and uneasiness in food ingestion [22]. Gastroparesis syndrome is characterized by tenderness in the upper part of the abdomen, including postprandial fullness, nausea, vomiting, and upper abdominal pain [23]. Indigested food changes lifestyles, and the depleted rate of healthcare directly affects the diversity of gut microflora. Fermented carbohydrates and diversified types of foods like stone fruits, lactose‐rich foods, and synthetic sweeteners are responsible for osmotic effects [24]. A gluten‐free diet is a very recommended diet for a celiac‐affected person [25].

At the molecular level, mutations in several genes disrupt normal signaling and bile acid receptor function, contributing to impaired gastrointestinal regulation. Notably, irritable bowel syndrome (IBS) has been linked to mutations in **SCN5A** and variations in **CDC42** and **NXP1**, with higher concordance observed among monozygotic twins [26, 27, 28]. However, microbial contamination results from pathogen infections, such as food poisoning and mutation (Table 1). Indigestion and constipation are common causative agents of *Clostridium*. Usually, they are caused by less fiber and water intake, excessive antibiotic consumption and even a lack of physical activity [29]. Dietary interventions have also been reported to alleviate constipation symptoms, though such approaches are better discussed in the context of traditional therapies. Diarrhea is one of the common water‐borne diseases caused by a variety of pathogens like *E. coli*, *Salmonella* spp., *Pseudomonas* spp., *Klebsiella* spp., *Campylobacter* spp., *Vibrio cholerae* , etc. Intestinal Worms: The two major phyla, that is, Platyhelminthes and Nematoda, are obligate parasites responsible for the cause of intestinal worms [30]. Traditionally, seeds of *Datura stramonium* and fruit decoction and leaves of *Melia azadirachta* are used against these worms and have been highly effective [31]. Additionally, dysentery is caused by *Entamoeba hystilica* and *shigella* and *E. coli* infection. These diseases are caused in areas with poor hygiene and affect the intestines of 25% of the world population; hence, they are considered the most infectious diseases after malaria [32, 33].

**TABLE 1:** List of some GI‐related pathogenic diseases, including causes, symptoms, and treatments [21, 22, 23, 24, 25, 26, 27, 28, 29, 30, 31, 32, 33].

Disease	Pathogenesis	Signs and symptoms	Diagnosis	Drugs/vaccine
Gastroesophageal reflux disease (GERD)	Decreased LES pressure due to genetic and environmental factors permits reflux of gastric contents.	Heartburn, hypersalivation, intermittent chest pain	X‐ray, endoscopy	Antacids, histamine blockers, proton pump inhibitors
Peptic ulcers	*Helicobacter pylori* infection activates neutrophils and macrophages, impairing mucosal defense and causing epithelial damage.	Nausea, bloating, loss of appetite, weight loss, blood in stool	Urea breath test, stool antigen assay	Amoxicillin, clarithromycin, tetracycline, metronidazole
Gastric adenocarcinoma	Chronic *H. pylori* infection, gastritis, genetic predisposition, and racial/geographic factors contribute to tumorigenesis.	Weight loss, abdominal pain, altered bowel habits, nausea	Endoscopic gastroduodenoscopy (EGD)	Capecitabine, carboplatin, docetaxel, oxaliplatin
Gastrointestinal stromal tumor (GIST)	Mutation in *c‐kit* gene activates RAS pathway, promoting abnormal cell proliferation.	Abdominal pain, bloating, nausea, weight loss	Endoscopy, colonoscopy, CT/MRI	Imatinib, sunitinib, ripretinib
Gastroenteritis	Inflammation of gastric and intestinal mucosa due to bacterial or viral infection.	Nausea, vomiting, abdominal pain, diarrhea, mild fever	Stool culture/test	Tetracycline, azithromycin
Salmonellosis	Pathogenic *Salmonella* invade intestinal mucosa after surviving gastric acid barrier.	Nausea, fever, diarrhea, abdominal pain, headache	Culture, serotyping, DNA fingerprinting	Fluoroquinolones, ampicillin
Malabsorption syndrome	Impaired nutrient absorption due to intestinal mucosal damage or enzyme deficiency.	Chronic diarrhea, bloating, weight loss, anemia	Stool test, nutrient absorption test	Nutritional supplementation
Inflammatory bowel disease (IBD)	Chronic immune response to gut flora causes mucosal inflammation and ulceration.	Diarrhea, fatigue, abdominal pain, bloody stool	Endoscopy	Infliximab, adalimumab, certolizumab, vedolizumab
Crohn's disease	Systemic inflammatory disease affecting the entire GI tract.	Crampy abdominal pain, diarrhea, fever	Intestinal endoscopy	Infliximab, adalimumab, certolizumab pegol
Ulcerative colitis	Chronic inflammation limited to colonic mucosa and submucosa.	Rectal pain, bleeding, diarrhea, weight loss	Intestinal endoscopy	Infliximab, adalimumab, golimumab
Rotavirus gastroenteritis	*Rotavirus* infection transmitted via fecal–oral route.	Fever, vomiting, severe diarrhea, dehydration	Stool enzyme immunoassay, RT‐PCR	Preventive vaccine (infants)

## 3 Traditional Approaches

GI disorders can disrupt normal digestion, absorption, and excretion processes. For centuries, medicinal plants have been widely used to manage these conditions due to their accessibility, affordability, and relatively low risk of side effects compared to synthetic drugs. In many rural regions, reliance on herbal medicine also reflects traditional knowledge systems and limited access to modern healthcare facilities [34, 35, 36, 37, 38, 39, 40].

Phytochemicals derived from plants exhibit diverse pharmacological actions including anti‐ulcerative, antidiarrheal, anti‐inflammatory, antispasmodic, and antimicrobial effects. Establishing scientific validation for these traditional practices not only preserves ethnomedicinal knowledge but also guides modern drug discovery and the development of bioactive compounds [41, 42, 43].

For instance, the ethanolic extract of *Albizia lebbeck* demonstrates antidiarrheal activity against *Vibrio cholerae* and *Aeromonas hydrophila* in animal models [44], while seeds of *Carum copticum* have long been used to alleviate diarrhea [45]. The active compound menthacarin from *Mentha piperita* (peppermint) relaxes intestinal smooth muscles by blocking calcium ion channels [46, 47, 48]. Similarly, gingerol from *Zingiber officinale* (ginger) acts on peripheral nerves to reduce gut hypersensitivity [49], and curcumin from *Curcuma longa* (turmeric) inhibits inflammatory mediators such as cyclooxygenase‐2 and TNF‐α, offering benefits for inflammatory bowel disease (IBD) and related disorders [50, 51, 52].

Other plants, such as *Cannabis sativa* , contain phytochemicals (notably Δ9‐tetrahydrocannabinol) that alleviate nausea, vomiting, and abdominal pain, though dosage and psychoactive effects require careful regulation [53, 54]. Collectively, these herbal treatments serve as complementary or supportive interventions that can reduce inflammation, restore gut motility, and enhance patient comfort when used alongside conventional medical therapies. However, overuse or unsupervised self‐medication can lead to toxicity or adverse interactions; hence, integration of traditional and clinical practices must be guided by scientific validation and safety assessments. Table 2 summarizes key medicinal plants by their therapeutic category, including anti‐ulcerative, antidiarrheal, and laxative effects.

**TABLE 2:** List various medicinal plants with their medicinal uses.

Therapeutic category	Plant species (common name)	Key bioactive compound(s)	Principal GI application(s)	References
Anti‐ulcerative agents	*Curcuma longa* (turmeric)	Curcumin	Inhibits COX‐2 and TNF‐α; reduces mucosal inflammation in IBD and ulcers	[50, 51, 52]
	*Kaempferia parviflora* (Thai ginseng)	Methoxyflavones	Protects gastric mucosa; reduces acidity and ulceration	[55]
	*Musa sapientum* (banana)	Polyphenols	Promotes mucosal healing; anti‐ulcer and antidiarrheal effects	[56]
Antidiarrheal and antimicrobial agents	*Acorus calamus* (sweet flag)	β‐asarone, acorin	Inhibits intestinal motility and pathogen growth	[57]
	*Albizia lebbeck* (siris tree)	Flavonoids, alkaloids	Active against *Vibrio cholerae* and *Aeromonas hydrophila*	[44]
	*Carum copticum* (ajwain)	Thymol, carvacrol	Relieves diarrhea and flatulence; antimicrobial	[45]
	*Centaurea solstitialis* (yellow star thistle)	Sesquiterpene lactones	Reduces intestinal motility and microbial infection	[58]
	*Elaeagnus angustifolia* (silver berry)	Phenolics	Controls acute diarrhea and gastritis	[59]
Laxatives and digestive stimulants	*Phyllanthus emblica* (amla)	Ascorbic acid, tannins	Natural laxative; enhances digestion	[60]
	*Tamarindus indica* (tamarind)	Tartaric acid	Mild laxative; relieves constipation	[61]
	*Ficus carica* (fig)	Ficin, pectin	Improves bowel movement; antispasmodic	[62]
	*Amygdalus communis* (almond)	Fatty acids, polyphenols	Laxative and digestive stimulant	[63]
Anti‐inflammatory and hepatoprotective agents	*Bombax ceiba* (red silk‐cotton tree)	Lupeol, gallic acid	Anti‐inflammatory; improves liver function	[64]
	*Mangifera indica* (mango)	Mangiferin	Anti‐inflammatory and antioxidant; supports hepatic and GI health	[65]
	*Oxalis corniculata* (wood sorrel)	Oxalic acid, flavonoids	Anti‐inflammatory, used for indigestion and piles	[66]
Anthelmintic and antiparasitic agents	*Alhagi camelorum* (camel thorn)	Alkaloids, flavones	Anthelmintic; relieves intestinal worm infections	[67]
	*Datura stramonium* (datura)	Tropane alkaloids	Used in traditional medicine for intestinal worms and constipation	[68]
Other notable agents	*Cannabis sativa* (cannabis)	Δ9‐tetrahydrocannabinol (THC)	Relieves abdominal pain, nausea, and vomiting; modulates gut motility	[53, 54]
	*Mentha piperita* (peppermint)	Menthacarin	Smooth muscle relaxant; reduces IBS‐related spasms	[46, 47, 48]
	*Zingiber officinale* (ginger)	Gingerol, shogaol	Antiemetic; modulates gut motility and reduces inflammation	[49]

## 4 Advanced Tissue Engineering Approaches

In the last few decades, several advanced treatments have been revealed for the treatment and complete functional recovery of damaged hollow organs such as the GI tract. The GI tract is generally partially or fully damaged, and infections are treated by either chemotherapies or surgical methods. These traditional medical practices are the most accepted clinical methods, but they lack proper functional recovery and result in poor life quality after surgery. Hence, innovative treatments have been formulated and designed to cure multiple types of GI disorders and regenerate functional tissue or organs (Table 3). These tissue regenerative and remodeling practices help to reconstruct the damaged portion of the GI tract by subsequent surgical grafting of bio‐engineered grafts and do not require repetitive, costly and painful treatment [91].

**TABLE 3:** Different tissue engineering approaches and their outcome in GI tract regeneration and remodeling.

Methods	Outcome	References
Xenogeneic biograft for the reconstruction of GI tract	Porcine SIS derived biodegradable graft applied for the healing of GI tract results better regeneration of damaged tissue without causing any post‐inflammatory rejection.	[69]
Wistar rats acellular esophagus in vitro cultured with esophageal epithelial cells	Within 1 week multilayered keratinized stratified three to four cellular thick epithelium layers form biomimetic to native tissue.	[70]
In vivo implantation of acellular esophagus matrix	Recellularized acellular matrix completely stratified epithelium cell layer vascualrized with insignificant inflammatory response post‐operative 30 days a thick keratin.	[71]
Acellular SIS bioscaffolds patch used to repair the damaged wounds	Complete regeneration of stratified esophagus tissue with restoration of function and no inflammation.	[72]
In vivo esophagus tissue regeneration using acellular matrix	Acellular gastric graft fabricated for the healing of in vivo esophagus wound in rat model. Complete tissue regenerated without any significant inflammation.	[73]
Studied the effect of acellular SIS scaffold for healing full‐thickness gastric wounds	Full‐thickness stomach wounds healed with the regeneration of gastric muscosal, smooth muscle and nerve cell formation. The regenerated tissue performs all the physiological activity.	[74]
Decellularized SIS recellularized with human stem cell in a bioreactor	Under in vitro condition intact muco‐vascularized ciliated tissue layer form.	[75]
Comparative study of different matrixes cultured with adipose‐derived mesenchymal stem cells	ECM matrix results enhanced differentiation of hepatocytes and expression of specific genes.	[76]
Primary rat hepatocytes in vitro cultured with the spleen and liver matrix	Spleen matrix showed better hepathocytic gene expression and functioning as compare to liver matrix.	[77]
Esophageal mucosa, SIS and UBM ECM	ECM derived from enhanced the migration of esophageal stem cells and promoted the formation of 3D organoids better than ECM derived from SIS or UBM.	[78]
PGA tubular scaffold seeded with epithelial cells along with the fibroblast cultured in collagen sheet	Recapitulate subepithelial fibroblast involvement in bioengineered esophagus or intestine.	[79]
Primary rat hepathocytes cultured with ECM hydrolyastes of different animals and human	Porcine and canine liver ECM showed higher secretion of albumin and bile, however human liver ECM have better outcome for primary human hepatocytes.	[80]
In vitro study of acellular SIS matrix recellularized with intestinal organoid having epithelial and progenitor cells	After 7 days incubation intact tissue layer of epithelial, fibroblast and other differentiated cells.	[81]
Studied the effect of 2D and 3D spheroid on stem cell differentiation into the specific hepatic cell lineage	Cell cultured on 3D spheroids liver ECM differentiated into specific cell lineage with gene expression.	[82]
Studied the effect of solubilized liver ECM on iPSC differentiation	Solubilized tissue specific ECM peptides result enhanced cell signaling, differentiation into the specific hepatic tissue cell lineages.	[83]
Collagen rings seeded with mouse intestinal organoids	Aligned epithelial lining form tubular structure but in situ inflammation and infection.	[84]
Bio‐engineered two stage esophageal graft consist of decellularized native esophagus tissue recultured with the mesoangioblasts and fibroblasts	Implantation of anatomically systematized and pre‐vascularized esophageal graft results better functional recovery of the regenerated.	[85]
Semi‐synthetic graft seeded with autologous conduit isolated from stomach, small bowel, or colon in vitro cultivated in a bioreactor system	Nanofibrous matrix seeded induced the growth of adipose‐derived mesenchymal stem cells (aMSCs) into the endogenous esophageal tissue layers with complete vascularization.	[86]
Two‐layered tubular scaffold and mesenchymal stem cell‐based bioreactor system	Circumferential esophageal repair is technically challenging in a rat model, more than 80% of the mucosal regeneration without a fistula, stratified squamous epithelium with several newly developed blood vessels.	[87]
Artificial intestinal graft poly(glycerol sebacate) (PGS)	In vitro and in vivo biomimics to the native tissue, have significant ultrastructure and physicochemical architecture for the treatment of short bowel syndrome.	[88]
Humanized (3D) co‐culture in vitro model of fibroblasts upon the Caco‐2 epithelium cellular layer	Endogenous extracellular matrix production from the fibroblasts by the direct contact between the epithelial and mesenchymal cells.	[89]
Collagen tube embedded with human duodenum organoids (HDOs)	Bio‐engineered gastric tissues contagion with *Helicobacter pylori* and represented as model for drug‐testing and pathological testing.	[90]

**FIGURE 2:**
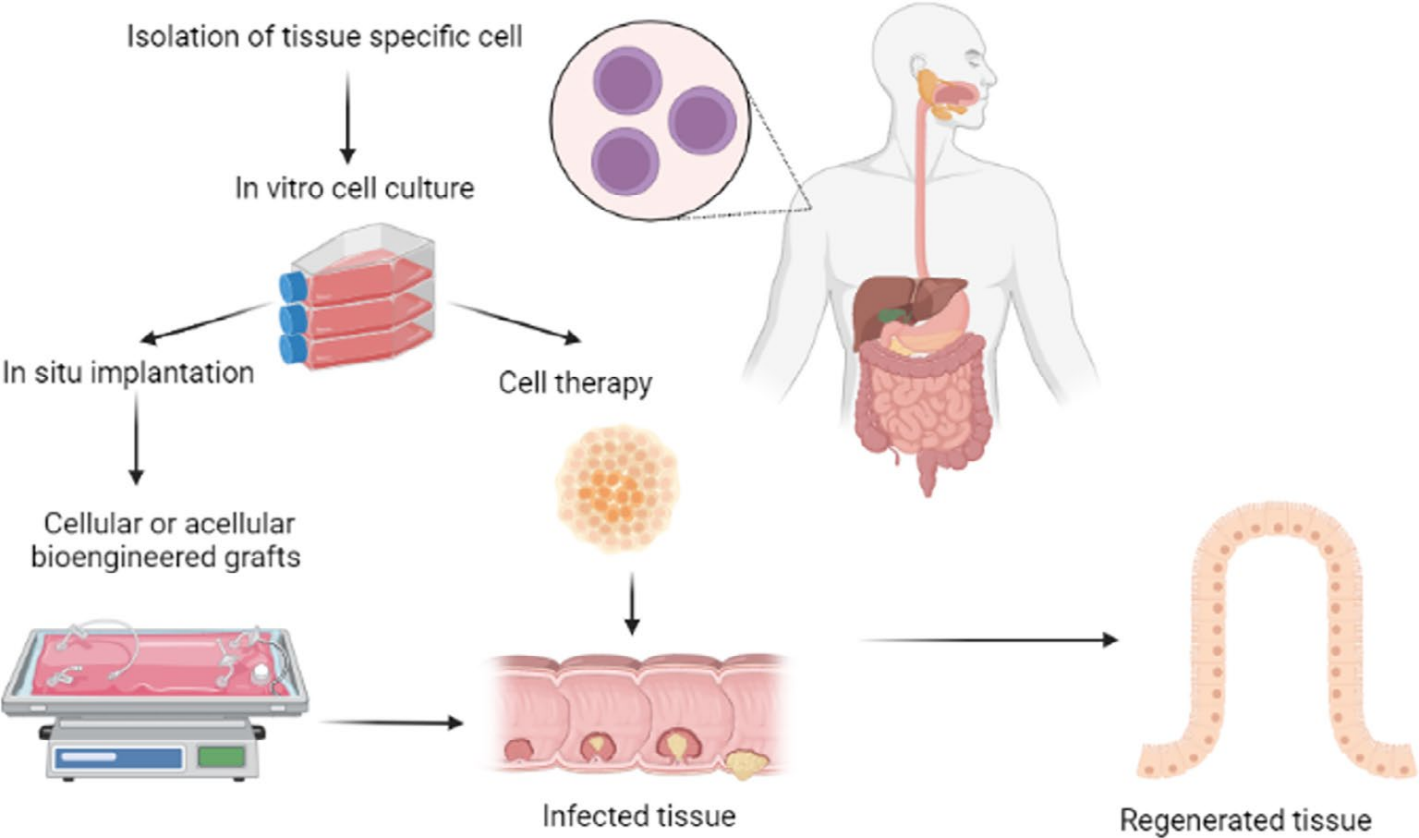
Outline of tissue engineering and regenerative medicine approaches for repairing and remodeling GI tract problems.

Tissue engineering and regenerative medicine are the most advanced techniques for designing bio‐engineered grafts to replace the damaged or distorted tissue/organ and regenerate the neo‐bio‐functional tissues (Figure 2). Then the bio‐engineered grafts of polymeric scaffolds and tissue‐specific cells or acellular are commercially available to reconstruct different body parts or organs [10, 11]. However, in the case of the GI tract, numerous efforts and experiments at the cellular to pre‐clinical level have been conducted by researchers. Still, the research has been ongoing to achieve success in reconstructing this complex cellular tissue and organization. Even different types of synthetic and natural polymers, their blends and composites are used to fabricate a whole tubular scaffold for GI tract repair, but multi‐layered stable tissue reconstruction is difficult [87]. Decellularized tissue having an intact 3D biomimetic, biocompatible structure as the native tissue provides better outcomes, but limited availability of donor tissue and laborious procedures are the main issues [12, 78].

**FIGURE 3:**
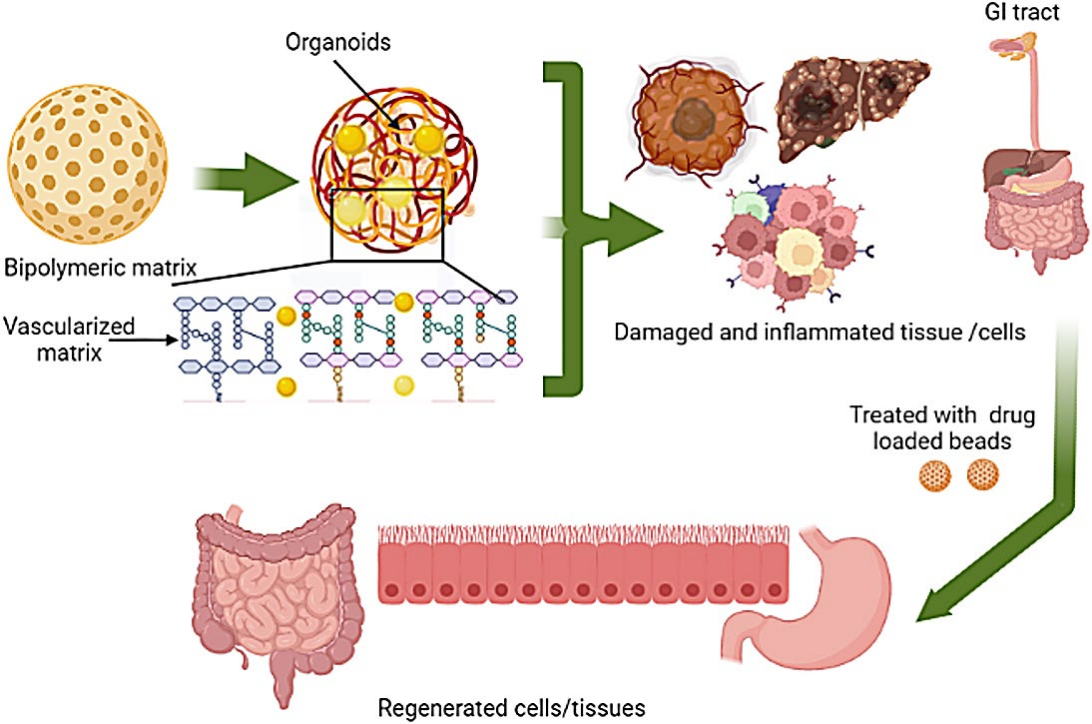
Organoids system synthesis and functioning of damaged and infected tissue or organs.

Recent studies have reported that the semi‐synthetic GI bio‐engineered construct of synthetic polymer and decellularized ECM provides promising outcomes in in vivo animal model systems. Different biomaterials have been studied and optimized to promote cell survival and differentiation. In vitro results do not necessarily translate into in vivo; therefore, animal studies must confirm the results in the correct animal models [11, 90]. Those units can be isolated from various gut parts and seeded onto scaffolds for implantation (Figure 3). The organ‐on‐a‐chip technique has been introduced to the field of GI regeneration. This highly sophisticated technique enables the study of the physiology and pathophysiology of the system [92]. This system can study the absorption of drugs, the interaction between the epithelium and the microbiome, and the barrier function of the epithelium.

Despite impressive laboratory success, clinical translation remains challenging. Regulatory approval for implantable biomaterials requires extensive biocompatibility testing, standardized manufacturing protocols, and long‐term safety evaluation. Cost and scalability issues also limit the commercial deployment of bioengineered GI grafts. Future directions should emphasize ethical compliance, cost‐effective fabrication, and integration of interdisciplinary expertise to ensure patient accessibility and clinical reliability.

## 5 Limitation and Future Prospectus

Despite major progress in GI tissue engineering, considerable technical and translational challenges remain. While in vitro experiments have demonstrated the successful development of scaffolds and organoids, these systems often fail to replicate the complex physiological environment of the human gut, where mechanical stress, peristaltic motion, vascularization, and immune responses determine functional success [93]. Limited mechanical stability, inadequate vascular integration, and inconsistent cell signaling within engineered grafts continue to restrict clinical applicability. Moreover, the translatability of preclinical data is hindered by the absence of standardized models and reproducible bioreactor systems. The use of xenogeneic extracellular matrices, although promising, carries risks of pathogen transmission and immunogenicity, underscoring the need for rigorous biosafety and ethical assessment [94, 95].

Recently, in vitro studies still need to be performed at in vivo or preclinical levels to investigate graft's in situ response under physiological conditions and their commercial application. Lack of preclinical and clinical trial data is the key limiting factor in repairing GI tracts. These systems enable precise evaluation of cellular interactions, drug absorption, and tissue remodeling, thereby bridging the gap between laboratory and clinical applications. Future research should focus on optimizing biomaterial composition for cost‐effective scalability, improving vascularization and long‐term graft function, and developing unified regulatory frameworks to ensure patient safety and translational reliability [96, 97, 98]. By integrating interdisciplinary advances in biomaterials, stem cell biology, and biomedical engineering, GI bioengineering is poised to evolve into a clinically functional, ethically compliant, and sustainable therapeutic domain.

## 6 Conclusion

We highlight traditional and advanced gastrointestinal tissue engineering approaches, emphasizing their technical aspects and societal benefits. Despite the availability of current treatments, many remain invasive, expensive, and often ineffective after prolonged surgical procedures. The integration of herbal bioactives, biocompatible scaffolds, and cell‐based regenerative techniques provides a promising alternative that can enhance healing, reduce inflammation, and promote natural tissue remodeling. Sustainable sourcing and validation of traditional medicinal compounds remain crucial to ensure the therapeutic safety and reproducibility of these compounds. Moving forward, interdisciplinary collaboration will be vital in transforming laboratory advances into clinically viable solutions. Optimizing biomaterial composition, improving vascularization, and adopting organoid and organ‐on‐chip systems will accelerate translational progress. Additionally, some plants may contain toxic compounds that could harm human health. Thus, carefully selecting materials for graft fabrication, cell sources for tissue regeneration, and controlled physiological conditions are essential for achieving complete tissue recovery. By combining expertise from biotechnology, materials science, and pharmacognosy, future research can bridge the gap between experimental innovation and real‐world application, ultimately offering more effective, affordable, and ethically responsible treatments for gastrointestinal disorders.

## Funding

The authors have nothing to report.

## Ethics Statement

The authors have nothing to report.

## Consent

The authors have nothing to report.

## Conflicts of Interest

The authors declare no conflicts of interest.

## Data Availability

The authors have nothing to report.
